# Editorial: The interaction of chronic viral infections and SARS-CoV-2 infection and its effect on the COVID-19 pathogenesis

**DOI:** 10.3389/fmicb.2025.1727446

**Published:** 2025-11-12

**Authors:** Hem Chandra Jha, Timsy Uppal, Prerna Dabral, Subhash C. Verma

**Affiliations:** 1 Mehta Family School of Biosciences and Biomedical Engineering, Infection Bio-Engineering Group, IIT Indore, Simrol, India; 2 Department of Microbiology and Immunology, University of Nevada, Reno School of Medicine, Reno, NV, United States; 3 Vitalant Research Institute, San Francisco, CA, United States; 4 University of California, San Francisco, San Francisco, CA, United States

**Keywords:** SARS-CoV-2, chronic viral infections, cytokine storm, antiviral, PASC, predictive modeling, neuroinvasion

## Introduction

The COVID-19 pandemic highlighted the intricate relationship between acute viral infections and pre-existing chronic viral conditions. Severe acute respiratory syndrome coronavirus 2 (SARS-CoV-2), besides triggering strong immune responses, interferes with the immune regulatory mechanisms of chronic illnesses, such as those caused by herpesviruses, hepatitis, and other chronic viruses. These interactions influence susceptibility, disease severity, and long-term outcomes, including post-acute sequelae of COVID-19 (PASC).

**Figure 1:**
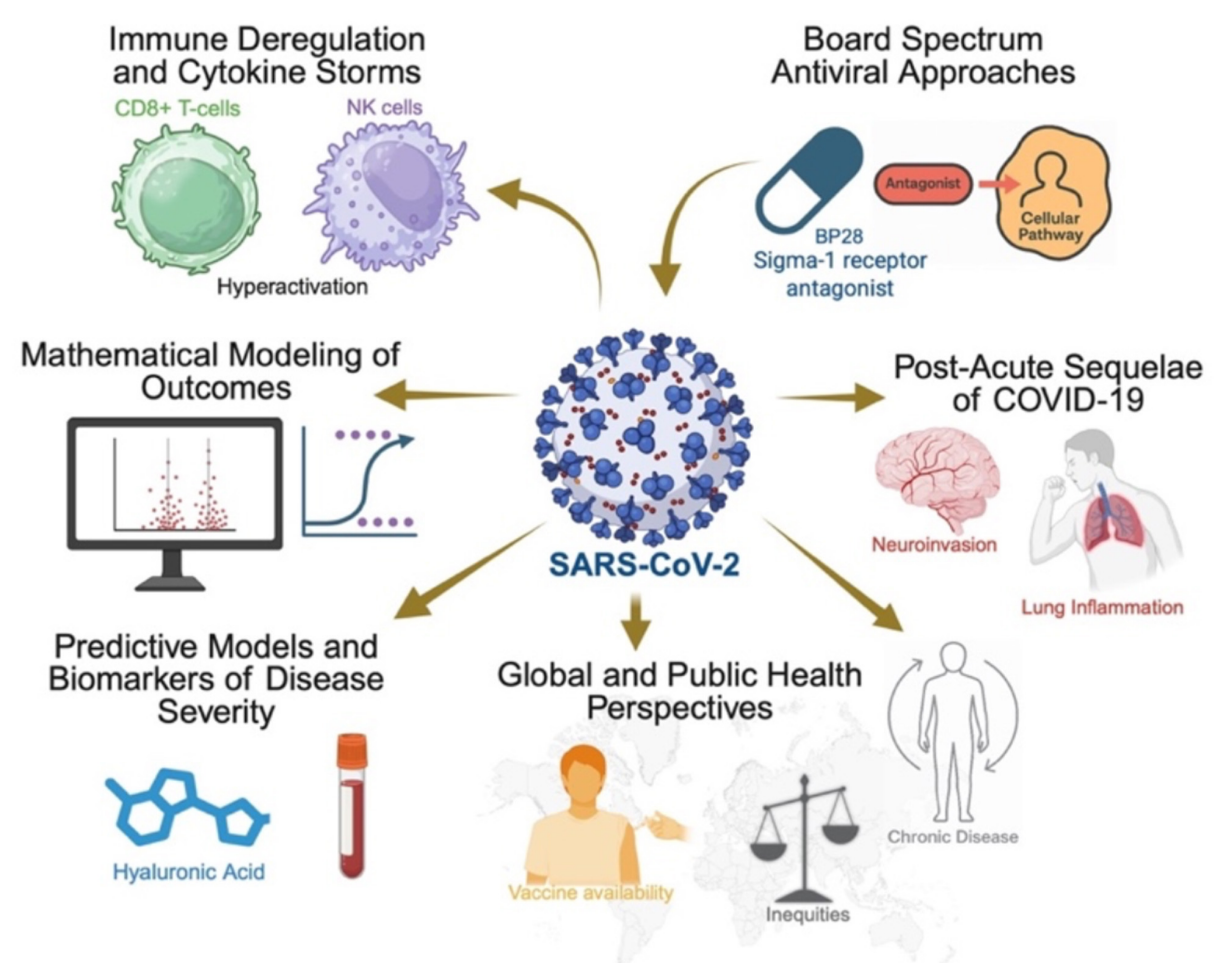
Integrative schematic of the Research Topic “*The interaction of chronic viral infections and SARS-CoV-2 infection and its effect on COVID-19 pathogenesis*.” This summarizes the key themes highlighted across the collected articles. Immune dysregulation and cytokine storms illustrate the paradox of CD8^+^ T cell exhaustion alongside hyperactivation of NK cells. Mathematical modeling of outcomes captures the heterogeneous trajectories of disease progression. Broad-spectrum antiviral approaches, such as the sigma-1 receptor antagonist PB28, represent host-targeted therapeutic strategies. Post-acute sequelae of COVID-19 include neuroinvasion and lung inflammation, linking acute infection with long-term consequences. Predictive models and biomarkers of disease severity emphasize the prognostic role of factors such as hyaluronic acid. Finally, global and public health perspectives stress the impact of inequities, vaccine availability, and chronic disease burdens on outcomes.

This Research Topic features contributions ranging from original research and reviews to hypothesis-driven modeling and opinion pieces. Together, these findings deepen our understanding of the connection between chronic viral infections and SARS-CoV-2, as well as how these insights inform therapeutic, diagnostic, and public health strategies. A schematic overview of the Research Topic is presented in Figure 1, summarizing the interconnections between chronic viral infections, immune dysregulation, SARS-CoV-2 pathogenesis, therapeutic strategies, and public-health determinants discussed throughout this issue.

## Immune dysregulation and cytokine storms

Meggyes et al. conducted a comprehensive immunophenotypic study of COVID-19 patients experiencing cytokine storms, demonstrating that severe cases were associated with lower levels of CD8^+^ T cells, accompanied by hyperactivation of CD8^+^, NK, and NKT cells, and increased expression of the PD-1/PD-L1 and TIGIT pathways. This shows simultaneous hyperactivation and exhaustion, which hinders viral clearance while exacerbating inflammatory damage. This dual phenotype provides a mechanistic explanation for immune checkpoint regulation in severe COVID-19, highlighting how chronic viral immune imprinting may exacerbate SARS-CoV-2 pathology.

## Broad-spectrum antiviral approaches

The search for pan-coronavirus therapeutics is crucial due to the rise of variants and co-infections. Song G. et al. showed that PB28, a sigma-1 receptor antagonist, has broad-spectrum antiviral activity against α-, β-, and γ-coronaviruses. In the lab, PB28 stopped the replication of SARS-CoV-2 (including Beta, Delta, and Omicron variants), HCoV-OC43, and animal coronaviruses. In live studies with mice and chicken embryos, it led to lower viral loads, less tissue damage, and higher survival rates. This Research Topic highlights host-targeted strategies as promising treatment options, particularly for patients with weakened immune systems resulting from chronic viral infections.

## Viral proteins as diagnostic and vaccine targets

Two comprehensive reviews in this Research Topic highlight the translational importance of viral proteins. Song W. et al. examined the nucleocapsid (N) protein as a target for diagnostics and vaccines. Unlike the spike protein, the N protein is highly conserved across SARS-CoV-2 variants. It mounts a strong antibody response, confirming this as a reliable biomarker for serological testing and a potential component of cross-variant vaccines. Li X. et al. provided an extensive review of SARS-CoV-2 pathogenesis, variants, and vaccines. This discusses the structural basis of immune escape, the role of cytokine storms in disease progression, and therapeutic strategies that target both viral replication and host inflammation. This highlights the importance of combining viral protein conservation with host immune modulation in the development of vaccines and therapeutics.

## Predictive models and biomarkers of disease severity

Yu and Huang developed a mechanistic model that simulates alveolar infection, viral replication, immune-mediated damage, and cellular regeneration to explain why COVID-19 outcomes vary widely. Their analysis revealed three distinct trajectories: abortive, self-limited, and severe infections, driven by the balance between healthy and damaged alveolar cells. It notes that once tissue injury surpasses a critical threshold, disease progression accelerates and therapeutic benefit diminishes, emphasizing the importance of early intervention. Yang et al. developed sex-specific predictive models for critical illness, identifying 12 male-specific and 10 female-specific risk factors, which indicate significantly higher mortality among older men. This stratification is vital in personalized medicine, emphasizing how biological sex, along with chronic viral immune legacies, affects COVID-19 outcomes. Similarly, Li Y. et al. discovered that hyaluronic acid (HA) functions as a prognostic biomarker, and its elevation can predict mortality in both primary and reinfected patients. However, HA was not associated with the development of long COVID. These findings imply that extracellular matrix remodeling and fibroproliferative pathways, which are already disrupted in chronic infections, play a crucial role in determining the severity of SARS-CoV-2 infection.

## Neurological and multisystem pathogenesis

Neurological symptoms of COVID-19 remain a key part of the disease's progression. Hsu et al. used AC70 human ACE2 transgenic mice to demonstrate that SARS-CoV-2 can infect the nervous system not only via the olfactory nerve but also through the trigeminal nerve. The viral spread throughout the brain altered neuronal gene expression related to dopamine and synaptic function, even in the absence of apparent tissue damage. This finding highlights the possibly overlooked routes of neuroinvasion and draws parallels with chronic neurotropic infections like HSV, where latent reservoirs affect CNS pathology.

## Opinion: syncytia and viral persistence

Palchevska and Dominguez presented a provocative opinion article suggesting that syncytia formation serves as a “viral escape room,” allowing SARS-CoV-2 to persist. They emphasize that syncytia, multinucleated giant cells formed through spike-mediated cell–cell fusion, support immune evasion, and may serve as virus reservoirs in long COVID. This concept situates SARS-CoV-2 within the broader category of persistent RNA viruses, aligning with observations that viral proteins persist in tissues for months after infection. The opinion prompts reconsideration of SARS-CoV-2 not just as an acute pathogen but as one capable of persistence through unconventional cellular mechanisms.

## Global and public health perspectives

Socioeconomic and structural factors are key determinants of COVID-19 outcomes. In Burkina Faso, Kamga et al. found that age, comorbidities, and urban residence were predictors of poor outcomes, and mortality rates remained largely unchanged despite the widespread use of hydroxychloroquine–azithromycin combinations. The findings align with large, randomized trials (Recovery Collaborative Group, 2020; WHO Solidarity Trial Consortium, 2021; Axfors et al., 2021), which have consistently shown that hydroxychloroquine offers no mortality benefit in hospitalized COVID-19 patients. Conversely, Arbel et al. demonstrated that population size, age distribution, socioeconomic status, and vaccine availability were primary factors influencing mortality trends in Israeli cities. Collectively, these studies emphasize that inequities and the burden of chronic diseases, rather than unproven therapies, significantly shape pandemic outcomes, underscoring the importance of evidence-based approaches and robust health systems.

## Integrative perspective

The insights from this Research Topic highlight three key themes that influence the relationship between chronic viral infections and COVID-19. First, immune imprinting caused by long-term infections has a significant impact on disease severity. Factors such as checkpoint exhaustion, ongoing immune activation, and changes in extracellular matrix biology demonstrate how immune systems affected by lifelong viral exposures respond to SARS-CoV-2 with increased vulnerability and dysregulated inflammation. Second, host-targeted therapies and conserved viral proteins are vital for future pandemic preparedness. The broad effectiveness of sigma-1 receptor antagonists, like PB28, along with the diagnostic and vaccine potential of the nucleocapsid protein, underscores an approach that goes beyond the limitations of spike-focused treatments and variant-specific immunity. Third, public health outcomes depend on both biological and structural factors. Sex-specific risk factors, hyaluronic acid as a prognostic marker, and socioeconomic disparities highlight the need for predictive models and intervention strategies that merge molecular, clinical, and demographic data.

## Conclusion

The findings of these studies demonstrate that COVID-19 cannot be fully understood in isolation but must be examined within the broader context of chronic viral infections and social factors that impact health. By combining insights from immunopathogenesis, therapeutic development, biomarker discovery, predictive modeling, and global epidemiology, these articles provide guidance for preparing for future pandemics. This highlights how chronic viral signatures influence the course of acute illness, treatment outcomes, and recovery, including the long-term effects of long COVID. Through original research, reviews, models, and opinions, this Research Topic delivers a key message: chronic viral infections can be a significant concern. Recognizing and addressing these interactions will be crucial for improving COVID-19 management and for predicting and reducing the impact of future viral outbreaks.

## References

[B1] Axfors C. Schmitt A. M. Janiaud P. Van't Hooft J. Abd-Elsalam S. Abdo E. F. . (2021). Mortality outcomes with hydroxychloroquine and chloroquine in COVID-19 from an international collaborative meta-analysis of randomized trials. Nat. Commun. 12:2349. doi: 10.1038/s41467-021-22446-z 33859192 PMC8050319

[B2] Recovery Collaborative Group (2020). Effect of hydroxychloroquine in hospitalized patients with COVID-19. New Engl. J. Med. 383, 2030–2040. doi: 10.1056/NEJMoa2022926 33031652 PMC7556338

[B3] WHO Solidarity Trial Consortium (2021). Repurposed antiviral drugs for Covid-19—interim WHO solidarity trial results. New Engl. J. Med. 384, 497–511. doi: 10.1056/NEJMoa2023184 33264556 PMC7727327

